# Extended Visual Sequence Learning Leaves a Local Trace in the Spontaneous EEG

**DOI:** 10.3389/fnins.2021.707828

**Published:** 2021-07-16

**Authors:** Serena Ricci, Elisa Tatti, Aaron B. Nelson, Priya Panday, Henry Chen, Giulio Tononi, Chiara Cirelli, M. Felice Ghilardi

**Affiliations:** ^1^Department of Physiology, Pharmacology and Neuroscience, CUNY School of Medicine, New York, NY, United States; ^2^Department of Informatics, Bioengineering, Robotics and Systems Engineering, University of Genoa, Genoa, Italy; ^3^Department of Psychiatry, University of Wisconsin-Madison, Madison, WI, United States

**Keywords:** learning, fatigue, homeostasis, visual learning, resting state EEG

## Abstract

We have previously demonstrated that, in rested subjects, extensive practice in a motor learning task increased both electroencephalographic (EEG) theta power in the areas involved in learning and improved the error rate in a motor test that shared similarities with the task. A nap normalized both EEG and performance changes. We now ascertain whether extensive visual declarative learning produces results similar to motor learning. Thus, during the morning, we recorded high-density EEG in well rested young healthy subjects that learned the order of different visual sequence task (VSEQ) for three one-hour blocks. Afterward, a group of subjects took a nap and another rested quietly. Between each VSEQ block, we recorded spontaneous EEG (sEEG) at rest and assessed performance in a motor test and a visual working memory test that shares similarities with VSEQ. We found that after the third block, VSEQ induced local theta power increases in the sEEG over a right temporo-parietal area that was engaged during the task. This local theta increase was preceded by increases in alpha and beta power over the same area and was paralleled by performance decline in the visual working memory test. Only after the nap, VSEQ learning rate improved and performance in the visual working memory test was restored, together with partial normalization of the local sEEG changes. These results suggest that intensive learning, like motor learning, produces local theta power increases, possibly reflecting local neuronal fatigue. Sleep may be necessary to resolve neuronal fatigue and its effects on learning and performance.

## Introduction

Intense learning and acquisition of novel experiences leave local traces in the brain activity that are evident at rest, after the performance, in the areas that were specifically involved in the learning-related processes. Such traces emerge in the spontaneous electroencephalogram during rest as local power increases in the theta range (Vyazovskiy et al., 2011b; Hung et al., 2013; Bernardi et al., 2015; Nelson et al., 2021). As global theta power surge during resting state can be considered as a marker of sleep need (Akerstedt and Gillberg, 1990; Cajochen et al., 1995, 1999; Vyazovskiy and Tobler, 2005), it is likely that the local theta increases represent neuronal tiredness caused by the local, cumulative cellular costs of synaptic plasticity associated with learning (Vyazovskiy et al., 2011b; Hung et al., 2013; Bernardi et al., 2015; Nelson et al., 2021). In fact, theta power increases locally after training in a specific task, in the areas involved in the learning, in both sleep-deprived subjects (Hung et al., 2013; Bernardi et al., 2015, 2016) and animals (Vyazovskiy et al., 2011b), but also in well rested subjects (Nelson et al., 2021). Indeed, spontaneous EEG (sEEG) recordings in well rested subjects showed power increases in the theta band (4–8 Hz) over a frontal cluster of electrodes after intensive learning with a visuo-motor adaptation task (Nelson et al., 2021). In agreement with the previous work in sleep-deprived subjects, the frontal theta increase may represent local sleep, defined as slow wave activity occurring locally in the awake brain (Huber et al., 2004), and induced by these areas engagement in learning- and plasticity-related processes (Hung et al., 2013; Bernardi et al., 2015). In fact, such a local increase was not present after a motor task lacking the learning component; it was associated with an increased error rate during tests involving the same motor regions, and it was reduced only after a nap and not by quiet wake.

In the present study, we ascertain in well-rested subjects whether intensive learning in a declarative task produces effects similar to those of the motor learning task both on the resting sEEG and on the performance of a test that shared task characteristics. We also determined whether a nap can reduce both local theta power increases and performance deterioration. To induce intensive declarative learning, we used a visual sequence task (VSEQ) where subjects learned the order of spatial sequences of targets. This task, which does not have any motor component, engages visual spatial attention and working memory (Ghilardi et al., 2003, 2009; Moisello et al., 2013) and is associated with EEG activity of the frontal and posterior parietal areas mostly in the right hemisphere (Moisello et al., 2013). The EEG pattern during the VSEQ task likely reflects encoding of new information, access to memory storage and activation of memory traces, in agreement with the results of studies on cognitive and semantic memory formation (Klimesch et al., 1993, 1997, 2011; Klimesch, 1997; Bastiaansen et al., 2002). Importantly, after learning for 20–30 min the order of a single sequence of targets in VSEQ, the post-task sEEG displayed a power increase in the alpha range over temporo-parietal regions where power changes occurred during the task (Moisello et al., 2013). Based on the results of the study with intensive visuo-motor learning (Nelson et al., 2021), one should expect a local increase of theta power with more than 1 h of performance in the VSEQ task. Also, the local theta power increase should be accompanied by a rise in performance errors in tests involving the neural circuits fatigued by VSEQ learning and should be abolished by subsequent sleep. Therefore, during the morning hours, we recorded EEG in a group of well rested subjects during extended performance of VSEQ in three one-hour blocks and during the sEEG at rest after each block. We also assessed their performance in two brief tests performed at the end of each block: *mem*, a test that, like VSEQ, involves attention/spatial working memory and the activity of fronto-occipito-temporal areas; and *mov*, an arm reaching test that does not encompass learning components and that mainly requires the activity of sensori-motor areas (Perfetti et al., 2010; Moisello et al., 2015; Nelson et al., 2017; Tatti et al., 2019). We finally ascertained whether, as it occurred for motor learning (Nelson et al., 2021), sEEG and performance changes were restored by a 90-min nap but not by an equivalent period of quiet wake.

## Materials and Methods

### Subjects

We tested 32 right-handed healthy participants (age range: 19 – 35 years, mean ± SD = 23.8 ± 4.2 years, 16 women). All subjects did not have any history of sleep or medical disorders and were asked to maintain consistent bed rise times and 7–8 h/night sleep and fill daily a sleep diary for 1 week before the experimental session. Moreover, they were asked to abstain from alcohol and caffeine-containing beverages starting the night before and throughout the experiment. Subjects were trained in the task and tests (see below) in the weeks before the experiment. The study was approved by the local Institutional Review Board (CUNY IRB 307402 2016) and all the subjects signed and IRB-approved consent form.

### Experimental Design

**FIGURE 1:**
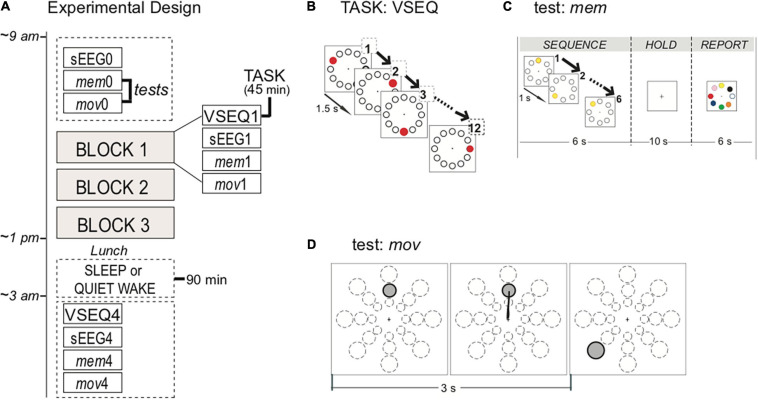
Experimental design, task, and tests. **(A)** Participants started the experiment at 9 am with baseline recordings of hd-EEG activity during 2 min of spontaneous EEG (sEEG), motor (*mov*) and memory (*mem*) tests. Then, subjects underwent three 45-min blocks of VSEQ task, each followed by sEEG, *mov* and *mem.* At the end of the morning, after a lunch break, a subset of subjects took a nap for 90 min, while the others quietly rested for the same amount of time. A further block of was performed 15–30 min after the rest. **(B)** VSEQ visual sequence learning task in which participants learned 12-element sequences continuously for 45 min. The same sequence was repeated until subjects reached full score. **(C)** *mem* is a visual working memory test without learning component. Instructions were to memorize a sequence, to hold it in memory for 10 s and then to report it, before moving to the next one; the test consisted in 16 sequences. **(D)** *mov* is a test of reaching movements without adaptation with 24 possible targets located at 4, 7, or 10 cm from the center in eight directions, that randomly appear on the screen every 3 s.

Subjects arrived at the lab by 8 am and were fitted with hd-EEG cap (256 channels, HydroCel Geodesic Net). Data collection started around 9 am and lasted until 3 pm (Figure 1A). Subjects were then seated in front of a screen and underwent a baseline recording that included 2-min sEEG with eyes opened and two tests, *mem* and *mov* (see section “Task and Tests”).

After the baseline, they performed VSEQ, where subjects were asked to learn visual sequences for 45 min, followed by a 2-min recording of sEEG with eyes open and by two tests, *mem* and *mov*. Each complete testing block lasted for about 1 h. At the end of the three complete blocks, 31 of the 32 subjects had lunch and were randomly assigned to one of two groups: nap (*N* = 14) or quiet wake condition (*N* = 17). The nap group was asked to sleep for 90 min; the quiet wake group was asked to rest with eyes closed while listening to a series of guided meditation and audiobooks for 90 min. After this 90-min period, the subjects in both groups performed a final block with sEEG, *mem*, *mov* and VSEQ. The final block was performed within fifteen-thirty minutes after the end of either the nap or the quiet wake.

### Task and Tests

VSEQ: Subjects were asked to learn 12-element spatial sequences presented on a screen (Ghilardi et al., 2003, 2009; Moisello et al., 2013; Steinemann et al., 2016) in 45-min blocks (Figure 1B). Twelve equidistant targets were displayed on the screen. Every 1.5 s one target randomly blackened for 300 ms in repeating sequences of twelve, with a target appearing only once in a sequence. A verbal report of the sequence order was collected every three complete presentations (i.e., a “*set*”) of the sequence. The same sequence was presented in repeated sets until fully learned, then a new one was presented. The learning rate for each block was computed as the average of the number of sets required to learn an individual sequence, an index of meta-learning, or “learning how to learn” sequences. The main point of this repetitive learning was to involve the same circuits for long time, while keeping the subjects engaged in the task.

*mem:* Subjects were asked to memorize a target sequence, to hold it in memory for 10 s after the presentation of the last target in the sequence, to report verbally the order of the sequence and to be ready for the next sequence (Figure 1C). A circular array of eight targets equidistant from a central point (4 cm) was present in the center of the screen. Targets were white circle outlined in black. After three warning flashes, the 5–6 elements sequence was presented with one target blackened for 250 ms followed by the others at 1-s intervals. Then, for 10 s the screen blanked (holding time) followed by the appearance of colored target array for 15 s (report time). Sixteen different sequences were presented for each of the four blocks. The outcome was the number of correct sequences per block. This test, which involves attention/spatial working memory, requires the activity of frontal and occipito-parietal areas like VSEQ (see: Supplementary Material and Supplementary Figures 1, 2).

*mov:* In this planar upper-limb reaching test, a target appeared on a screen in non-repeating, unpredictable order at 3-s interval together with a central starting point. Targets were at three different distances (4, 7, and 10 cm) and eight directions (45° separation). Subjects were asked to perform out and back overlapping movements, reaching for targets as soon and as fast as possible, but without anticipating or guessing the target position (Figure 1D). We excluded from the analysis movements whose parameters exceed 1.5 standard deviation of the mean. We computed the percentage of correct movements for each block. As previously reported, *mov* mainly involves the activation of the sensori-motor areas (Ghilardi et al., 2000, 2003; Perfetti et al., 2011; Tatti et al., 2019, 2020).

In general, the two tests lasted for less than 5 min and were designed to verify the effects of intensive repetitive learning in short tests, one involving similar circuits (*mem*) and the other involving also areas not engaged in the task such as the motor areas (*mov*).

### EEG Recordings and Analyses

During the whole experiment we recorded hd-EEG (Net Amp 300 amplifier and Net Station 5.0 software) maintaining the impedance of each channel below 50 kΩ. The sampling frequency of the EEG signal was 250 Hz and the signal was referenced to the vertex Cz. The EEG signal was filtered using a Finite Impulse Response filter (FIR) between 1 and 80 Hz and a Notch filter centered at 60 Hz. Then, the recording was divided into 4-s epochs and visually examined to remove artifactual epochs and channels. Additionally, we applied Independent Component Analysis (ICA) with Principal Component Analysis (PCA)-based dimension reduction (Jung et al., 2003) in order to remove blinks, eye movements and motion-related signals. Afterward, channels previously removed were interpolated using spherical spline interpolation and electrodes located on the face and neck were removed from the analyses. This results in 180 channels which were averaged referenced before being analyzed. All the preprocessing steps were processed using EEGLAB toolbox for Matlab (Delorme and Makeig, 2004). After the preprocessing, due to low-quality of the signal, we excluded the recordings of three subjects from the analysis of the EEG recorded during the VSEQ task.

We first computed power-spectral representations using the fast-Fourier transform function of FiedlTrip toolbox for Matlab (Oostenveld et al., 2011). Task-related EEG signal was normalized by the total EEG power of the 45-min session within five frequency ranges: Slow Wave Activity (SWA) or Delta: 1 – 4 Hz; Theta: 4.5 – 8 Hz; Alpha: 8.5 – 13 Hz; Beta: 13.5 – 25 Hz; Gamma: 25.5 – 35 Hz; while sEEG was normalized (i.e., subtracted and divided) by the baseline, i.e., the average across all the electrodes of the first sEEG recording at the beginning of the experiment in each frequency band.

We then determined: (i) practice-related activity, defined as the topographical EEG difference between the last and first learned sequences of VSEQ1; (ii) sEEG differences of morning blocks (sEEG1, 2, and 3) compared to the baseline (sEEG0). By using cluster-based non-parametric permutation testing (see Statistical Analysis), we identified clusters of electrodes showing significant differences between the two conditions, that we used as mask to determine personalized Regions of Interest (ROI). In fact, for each subject, we selected the electrode with the highest theta power in sEEG3 and its six closest neighbors within the sEEG3 clusters in order to assess differences between the nap and the quiet wake group, after the rest period.

Electroencephalographic data recorded during the nap and the quiet rest period were scored to detect signs of sleep using an open source Matlab toolbox (Mensen et al., 2016). Specifically, we scored 30-s epochs of EEG recording, according to standard guidelines (Iber et al., 2007). Transition from wakefulness (W) to stage N1 were associated with the disappearance of rhythms such as posterior alpha oscillations (8–10 Hz) and slow rolling eye movements. K complexes and sleep spindles marked the transition to N2. The transition and maintenance of N3 was determined by occurrence of >75 μV slow waves for more than 20% of the epoch. No REM sleep was detected in our recording. Sleep scoring was performed on classical derivations from the 10–20 montage (F4, F3, C4, C3, P3, P4, O1, O2), with a mastoid reference.

### Statistical Analysis

Within group differences in performance indices were evaluated between two time points and tested with two-tailed paired *t*-tests since all the difference distributions did not significantly deviate from normal distribution (*p* values > 0.1), as tested by Shapiro–Wilks tests (SW), followed by Kolmogorov Smirnov tests. In the following description, for each test we report *p* value for SW tests. We first compared learning rate at baseline to that at the end of the morning in the VSEQ task (VSEQ1 vs. VSEQ3, SW: *p* = 0.36) as well as test performance in *mov* (correct movements, *mov*3 vs. *mov*0, SW: *p* = 0.40) and *mem* (correct sequences, *mem*3 vs. *mem*0, SW: *p* = 0.63). This approach was also used to verify the within-group effects, for the nap and quiet wake groups separately, of a nap and quiet wake on task (learning rate in VSEQ3 vs. VSEQ4, SW: nap group: *p* = 0.61, quiet wake group: *p* = 0.47); to compare test performance (*mov*3 vs. *mov*4, SW: nap group: *p* = 0.35; quiet wake group: *p* = 0.72; and *mem*3 vs. *mem* 4 SW: nap group *p* = 0.48; quiet wake group: *p* = 0.30) and also to ascertain the effect of sleep on sEEG4 (SW nap group: delta or SWA: *p* = 0.28; theta: *p* = 0.41; alpha: *p* = 0.22; beta: *p* = 0.41; SW quiet wake group: delta *p* = 0.13; theta *p* = 0.34; alpha *p* = 0.32; beta *p* = 0.14).

EEG changes have been assessed through cluster-based non-parametric permutation testing. Specifically, nearest neighbor channels were determined via triangulation with three as the minimum number of significant channels for inclusion in a cluster. The reference distribution was created using Monte Carlo method with 10,000 random iterations and a critical alpha of 0.05 was used at the cluster level (Maris and Oostenveld, 2007).

Paired *t*-tests with Bonferroni correction for multiple comparisons were also used to compare spectral differences between sEEG0 and sEEG3 in the 5 frequency ranges. For all significant results with *t*-tests, we also computed effect sizes with Cohen’s d. Pearson coefficients (with Bonferroni corrections when appropriate) were used to explore possible correlations between: (1) performance measures and sEEG changes; (2) local power changes occurring during VSEQ1 and in the sEEG; (3) sEEG power changes after nap and sleep parameters; (4) performance changes and sleep parameters.

## Results

### Extensive Learning in a Visual Sequence Learning Task Produces Performance Changes and Leaves a Local Trace in the sEEG

In the three morning blocks of VSEQ, subjects learned an average of 27.35 (±SD. 1.24) 12-element visual sequences. Learning rate improved in that the average number of set presentations required to learn a single sequence significantly decreased from 3.02 ± SD. 0.79 in VSEQ1 to 2.63 ± SD. 0.74 in VSEQ3 (*N* = 32, two-tailed *t*-test: *t*(31) = 4.08, *p* = 0.0003, 95% CI: [0.15, 0.45], Cohen *D* = 0.93). These results suggest that subjects improved their skill in sequence acquisition with practice, in agreement with previous results (Marinelli et al., 2017).

**FIGURE 2:**
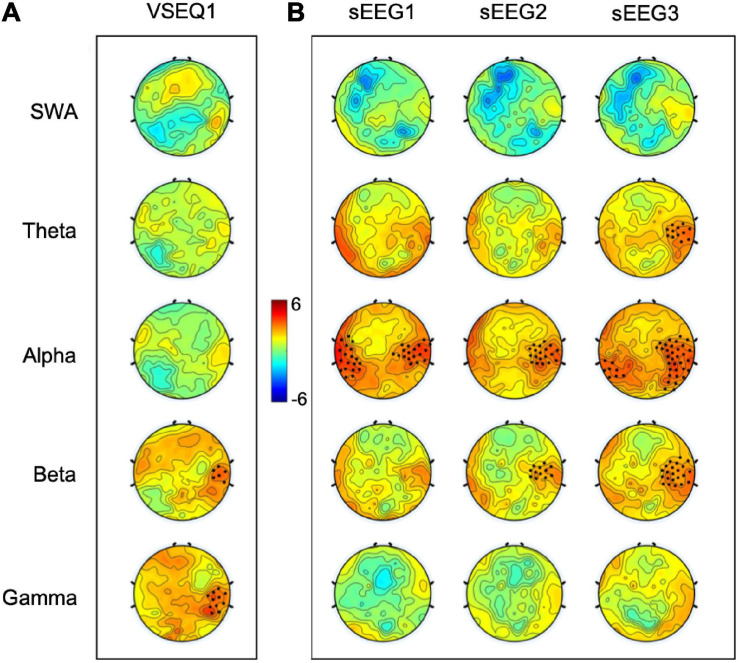
T-maps of the comparison between the last and first VSEQ1 sequences learned by the participants **(A)** and between sEEG activity recorded after each VSEQ block and sEEG0 baseline recording **(B)** in five frequency ranges. Black dots highlighted significant clusters of electrodes (*p* < 0.05, non-parametric cluster-based permutation testing).

We then determined the EEG correlates of practice in sequence learning by comparing the recordings obtained in the sets of the last sequence (average number of sets ± SD: 3.02 ± 1.20) and the sets of the first sequence (3.24 ± 1.56) of VSEQ1. We focused on VSEQ1 in order to minimize the effects of fatigue that might have accumulated across blocks. Cluster-based non-parametric permutation analysis (see section “Materials and Methods”) in the 1–55 Hz broad band did not show any significant clusters of power change. However, when we performed cluster analysis using conventional partition bands, we found a significant increase in both the beta (Hz, *N* = 29, mean ± SE: 10 ± 3%, cluster *t* = 18.86, *p* = 0.008) and gamma ranges (*N* = 29, mean ± SE: 14 ± 4%, cluster *t* = 35.39, *p* = 0.009) in a cluster of electrodes over the right temporo-parietal region (Figure 2A) that is usually active during visual working memory tasks (Berryhill and Olson, 2008).

**FIGURE 3:**
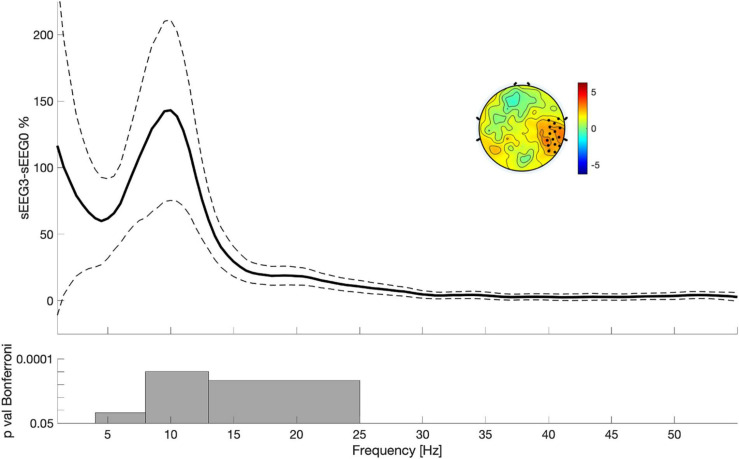
Power differences between sEEG3 and sEEG0 considering the electrodes highlighted in the topography. Lines indicate mean across all subjects (filled lines) and standard error of the mean (dotted lines). The topography is the result of the comparison between sEEG3 and sEEG0 in the entire spectrum (1–55 Hz). Black dots highlighted significant clusters of electrodes (*p* < 0.05, non-parametric cluster-based permutation testing). Bottom: results of the Paired *t*-tests with Bonferroni correction.

To ascertain whether VSEQ learning left a broad band (1–55 Hz) trace in the sEEG at the end of the morning, we compared sEEG3 and sEEG0 with cluster analysis. This analysis revealed a significant power increase in a set of electrodes over the right temporo-parietal area (mean ± SE: 26 ± 8%, cluster *t* = 47.24, *p* = 0.005, Figure 3). Such local increase was significant in the frequency ranges from theta to beta (from 4.5 to 25 Hz, Figure 3). Separate analyses for theta, alpha and beta frequency ranges (Figure 2B) confirmed that this increase involved a similar cluster of electrodes over the right area shown in Figure 2 (theta: mean ± SE: 44 ± 11%, cluster *t* = 39.89, *p* = 0.008; alpha: 62 ± 15%, cluster *t* = 165.73, *p* = 0.002; beta: 37 ± 10%, cluster *t* = 73.17, *p* = 0.004). We then asked whether these changes were already and equally present during sEEG1 and sEEG2. Power increased over the same area, but in a smaller cluster, in alpha range in both sEEG1 (28 ± 6%, cluster *t* = 122.24, *p* = 0.002) and sEEG2 (27 ± 11%, cluster *t* = 43.75, *p* = 0.004) and in beta range only in sEEG2 (20 ± 7%, cluster *t* = 28.93, *p* = 0.003; Figure 2B). Another cluster of power increase confined to the alpha range was found over a left temporo-parietal area area in sEEG1 (29 ± 6%; cluster *t* = 105.51, *p* = 0.002) and sEEG3 (36 ± 9%; cluster *t* = 119.45, *p* = 0.002; Figure 2B) but not in sEEG2, despite an average power increase of 33%, probably because of greater inter-subject variability (S.E. 11%). No significant clusters were found for the theta band in either sEEG1 and sEEG2, suggesting that the local theta power increase occurred only after a substantial power buildup in alpha and beta frequencies.

This conclusion is supported by the finding that theta power increase in the sEEG3 cluster moderately and positively correlated with the local power increases in both alpha range (*r* = 0.61, *p* = 0.0002, 95% CI for r [0.33, 0.79]; Supplementary Figure 3) and beta range (*r* = 0.71, *p* = 0.000005, 95% CI for *r* [0.49, 0.85]; Supplementary Figure 3) during sEEG2 singly and in combination (from 8 to 25 Hz, *r* = 0.73, *p* = 0.000002, 95% CI for *r* [0.52, 0.86]; Supplementary Figure 3).

The local increase in sEEG3 power in the theta range could signal the occurrence of neuronal OFF periods that has been associated to fatigue of the neural circuits involved in learning (Vyazovskiy et al., 2011b). In that case, after intensive *VSEQ* training, performance should deteriorate in *mem*, a working memory test sharing neural circuits and characteristics with VSEQ (Supplementary Figures 1, 2). Indeed, that was the case as the number of correctly reported sequences after VSEQ3 in *mem*3 slightly but significantly decreased compared to *mem*0 at baseline (mean ± SD: *mem*0: 12.81 ± 2.0; *mem*3: 12.03 ± 2.6; two-tailed *t*-test: *t*(31) = 2.054, *p* = 0.048, 95% CI: [0.01, 1.56], Cohen *D* = 0.40). The performance deterioration was specific to *mem*, as it did not occur in *mov*3, a motor test with little or no involvement of attention and spatial working memory (% correct movements: *mov*0: 80.67 ± 6.90%; *mov*3: 80.42 ± 8.95%; *t*(31) = −0.349, *p* = 0.85, 95% CI: [−1.67, 1.21]).

Altogether, these results suggest that intensive repetitive VSEQ training leaves a local trace to areas involved in the learning process and is associated with performance deterioration in a homologous test but not in a test involving other brain areas. Interestingly, intensive VSEQ learning also led to increases in subjective scores of tiredness (*N* = 32, Baseline: 3.69 ± 1.96; post VSEQ3: 5.62 ± 2.34, *t*(31) = −4.40, *p* = 0.0001, 95% CI [−2.84, −1.04], Cohen *D* = 0.78) but not of sleepiness (*N* = 32, Baseline: 4.62 ± 2.00; post VSEQ3: 5.00 ± 2.12, *t*(31) = −0.907, *p* = 0.37, 95% CI [−1.22, 0.47]) and boredom (*N* = 32, Baseline: 4.22 ± 2.11; post VSEQ3: 4.87 ± 2.00, *t*(31) = −1.96, *p* = 0.06, 95% CI [−1.34, 0.27]).

### A 90-min Nap Restores Performance in Mem and Improves Learning Ability in VSEQ

The results of the sleep scoring of the EEG signal during the 90-min rest period are reported in Supplementary Tables 1, 2. Briefly, the nap group slept on average 56 min (± S.E. 6), with most of the time spent in N2 and N3, thus suggesting that, in this group, sleep was consolidated and deep. Despite the instructions given to stay awake to the subjects in the quiet awake group, a few subjects reached N2 for a brief period of time (6 ± 2 min).

We first ascertained whether performance in the two tests, *mem* and *mov*, and in VSEQ improved after the 90 min of quiet rest and sleep. While performance in *mov* did not change significantly after the nap (*mov*3: 81.59 ± 4.69%; *mov*4: 80.38 ± 7.40%, *t*(11) = 0.46, *p* = 0.66, 95% CI: [−4.65, 7.08]), we found significant improvements in the nap group when comparing *mem*4 to *mem*3 (mean ± SD: *mem*3: 11.83 ± 2.37; *mem*4: 13.25 ± 1.96; *t*(11) = −3.137, *p* = 0.009, 95% CI: [−2.41, −0.42], Cohen *D* = 0.91). The positive effects of the nap were also extended to the ability to learn sequences during VSEQ. Indeed, the number of repetitions required to learn a sequence in the task slightly decreased in VSEQ4 compared to VSEQ3 after the nap (VSEQ3: 2.70 ± 0.72, VSEQ 4: 2.49 ± 0.63, *t*(11) = 2.64, *p* = 0.023, 95% CI: [0.35, 0.38], Cohen *D* = 0.76).

No performance changes were found after the quiet wake period in both *mem*4 (*mem*3: 12.31 ± 1.93; *mem*4: 12.54 ± 2.47; *t*(11) = −0.349, *p* = 0.733, 95% CI: [−1.67, 1.21]) and *mov*4 (*mov3*: 82.58 ± 7.19%; *mov*4: 80.13 ± 10.73%, *t*(11) = 0.072, *p* = 1.23, 95% CI: [−1.93, 6.82]), as well as in the VSEQ4 learning rate (VSEQ3: 2.61 ± 0.48, VSEQ 4: 2.73 ± 0.53, *t*(11) = −0.96, *p* = 0.36, 95% CI: [−0.39, 0.15]).

We then assessed possible relationship between performance improvements and SWA and theta power (from 1 to 8 Hz frequency) during sleep. Such analyses did not yield significant results between *mem* improvements and 1–8 Hz power of N2 and N3 combined (*N* = 12; *r* = 0.41, *p* = 0.19). Nevertheless, we found robust correlations between *mem*4 performance improvement and the power of the 1–8 Hz frequency range only during N3, in that the greater the 1–8 Hz frequency power during N3 the greater the increase of correct sequences in *mem*4 compared to *mem*3 both in the right cluster defined by the increased theta power in sEEG3 (*N* = 8, *r* = 0.857, *p* = 0.006595%, CI: [1.72, 9.38]) and across all electrodes (*r* = 0.947, *p* = 0.00035, 95%, CI: [3.16, 6.39]). Improvements of learning efficiency in VSEQ4 was moderately correlated with local theta power measured during N2 and N3 combined, in that the greater theta power during N2 and N3 the faster the learning in VSEQ4 compared to VSEQ3 (*N* = 12, *r* = 0.623, *p* = 0.030, 95% CI: [−0.72, −0.04]).

**FIGURE 4:**
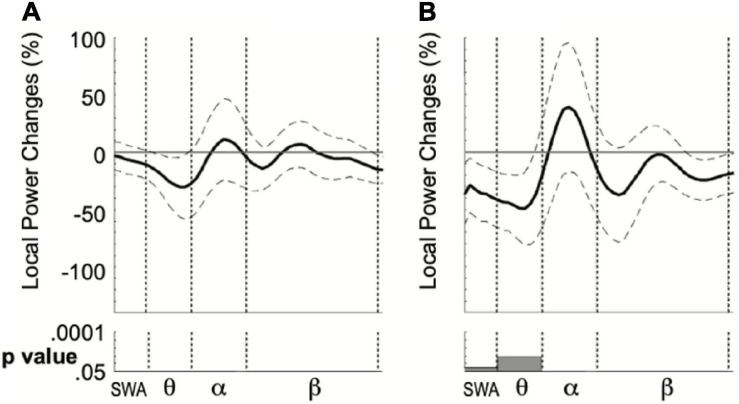
Local mean power difference between sEEG4 and sEEG3 considering the personalized ROI in the quiet wake **(A)** and nap **(B)** group. Dotted lines indicate standard error of the mean. Bottom: results of the Paired *t*-tests computed for four frequency ranges.

We finally computed the local power change in sEEG4 compared to sEEG3 in the right cluster of electrodes where theta power increase in sEEG3 was maximal. In the nap group, such comparison revealed a decrease in sEEG4 of theta frequency (*t* = −2.41, *p* = 0.031, 95% CI [−96.4%; −5.4%], Cohen’s *D* = 0.645). No significant changes were found in the other frequencies with the exception of a small mean decrease of SWA power (*t* = −2.21, *p* = 0.046, 95% CI [−69.9%; −0.8%], Cohen’s *D* = 0.59; Figure 4). In the awake group, no significant power changes were found across frequencies including (*t* = −1.01, *p* = 0.33; 95% CI [−24.5 8.69], Cohen’s *D* = 0.25) and theta (*t* = −1.38, *p* = 0.19; 95% CI [−68.8%; 14.5%], Cohen’s *D* = 0.30; Figure 4).

## Discussion

The present study shows that extensive declarative learning in well-rested subjects leaves a local trace over the brain regions involved in the task. This trace consists of a local power increase that, after three practice blocks, involves the theta frequency range. In parallel, learning rate in the VSEQ task increased, while performance in a test, *mem*, that shares some processes and neural substrates with VSEQ decayed. Performance in both the learning task and the working memory test improves after a nap but not after an equivalent period of quiet wake and the degree of improvement correlates to the local theta power occurring during the nap. Also, after the nap, local sEEG changes were partly renormalized. Altogether, these results first, suggest that, like motor learning, declarative learning produces local sEEG changes that may reflect neuronal fatigue, but, unlike motor learning, the significant theta power increase occurs in a cluster of electrodes over a right temporo-parietal area instead of left frontal region (Nelson et al., 2021). Second, they confirm that sleep may be necessary to resolve neuronal fatigue and its effects on learning and performance.

### Extensive Learning in a Visuo-Spatial Declarative Task Is Associated With Local Power Increases of Beta and Gamma During the Task

Visual sequence task and *mem* were characterized by similar topographical activation (Supplementary Figures 1, 2), have common attentional and working memory processes with similar spatial attributes and declarative characteristics related to order acquisition. However, while in *mem* the emphasis is mostly on encoding, with focused attention and working memory with sequences presented only once at a fast rate, in VSEQ, the focus is more on a continuous learning of complex sequences, one after the other, across several reiterations, thus requiring constant encoding and retrieval processes.

The initial phase in both VSEQ and *mem* encompasses attending for the target appearance, directing visual attention, processing a spatial position to then encode it. The efficiency of the spatial working memory buffer is crucial, in both VSEQ and *mem*, for linking one target’s position to that of the others and, in VSEQ only, for correctly predicting each target appearance and for checking the accuracy of each prediction in subsequent reiterations. Theta synchronization occurs in the early encoding phases of information and is considered as a hallmark of the cortico-hippocampal interplay at the cortical end (Sederberg et al., 2003; Pignatelli et al., 2012; White et al., 2013). Accordingly, in both VSEQ and *mem*, theta and alpha power modulation over centro-frontal and posterior regions, especially on the right hemisphere, occur during sequence learning [(Moisello et al., 2013), see also Supplementary Figure 1].

In VSEQ, we also found evidence of meta-learning or increased ability in “how to learn sequences.” Indeed, despite the training before the experiment, learning efficiency improved, with fewer sets needed to learn a sequence in VSEQ3 compared to VSEQ1. This suggests an enhancement of the use of active encoding processes primarily involving spatial working memory. The increased power in the beta and gamma bands over the temporo-parietal area during the learning block supports this conclusion in agreement with previous findings about gamma frequency activity in this area (Kaiser and Lutzenberger, 2005; Crone et al., 2006; Womelsdorf and Fries, 2006; Jerbi et al., 2009) also during visual working memory tasks (Baddeley, 1992; Smith and Jonides, 1997; Berryhill and Olson, 2008). Indeed, the temporo-parietal area can be considered as a hub where both spatial and object-related characteristics are processed and maintained. The simultaneous processing of both the “where” and the temporal order information is essential for successful VSEQ performance. Furthermore, the prevalent activity of the right hemisphere is in line with the results of imaging and electrophysiological studies (Moisello et al., 2013) and with the notion that spatial working memory tasks predominantly activate areas in the right hemisphere (Milner, 1971; Grafton et al., 1992; Jonides et al., 1993; McIntosh et al., 1994; Fink et al., 1996; Haxby et al., 1996; Ghilardi et al., 2000).

### Extensive Visual Sequence Learning Produces a Local Trace in the sEEG

Over these same electrodes showing beta and gamma increases during the task, we found a progressive local increase of alpha and beta power in the sEEG recorded during resting state, with a later involvement of theta power after VSEQ3 (Figure 2). The increase in the alpha range after VSEQ1 is in accordance with previous results where the same 16-element sequence was learned over twenty-thirty minutes (Moisello et al., 2013). Interestingly, the local increase of theta power at rest, after the third block of VSEQ was positively correlated to the earlier increases of both alpha and beta power over the same electrodes, suggesting that the involvement of lower frequencies occurs only after extending activity and a substantial build-up of higher frequency power. Indeed, the local increase in theta power is in agreement with the results of sleep deprivation studies (Hung et al., 2013; Bernardi et al., 2015), where local theta increases occurred with less intensive training only after 24 h of wake, as well as in well rested subjects after extensive motor learning (Nelson et al., 2021). Global increase of theta power during rest is an established hallmark of sleep need in both humans and animals, with EEG power in the 5–8 Hz range increasing globally with the time spent awake and predicting the SWA level in subsequent sleep (Akerstedt and Gillberg, 1990; Cajochen et al., 1995, 1999; Vyazovskiy and Tobler, 2005; De Gennaro et al., 2007). Local, as opposed to global, increases of sEEG theta power have been found over areas previously involved in learning in both animals (Vyazovskiy et al., 2011b) and humans (Hung et al., 2013; Bernardi et al., 2015) after 20–24 h of wake and, more recently, in well rested subjects after extensive learning in a visuo-motor adaptation task (Nelson et al., 2021). Increases of theta power during rest are likely expression of activity-related synaptic “overload” that, in turn, may cause neuronal instability and thus represent a sort of off-line signal (Klimesch, 1999; Vyazovskiy et al., 2011a). The preceding local increases in the alpha and beta range may thus reflect phenomena that bring to the build-up of synaptic overloading, such as increased neuronal firing and energy consumption (Ghilardi et al., 2021).

In summary, although parsimoniously it may simply reflect the massive changes in sEEG3, this local increase of theta power, or “local sleep,” could be interpreted as evidence of learning-related neuronal tiredness, in agreement with the conclusions of studies in rodents (Vyazovskiy et al., 2011b; Rodriguez et al., 2016) and in intracranially implanted epileptic subjects (Nir et al., 2017).

### Prolonged Visual Sequence Learning Causes Performance Deterioration Only in Specific Tests

Across the morning blocks, we observed two apparently conflicting phenomena: on one side, an improvement in the learning rate during VSEQ; on the other, a weakened performance in *mem*, a test sharing some characteristics and neural basis with VSEQ as discussed above. With repetitive practice and training, effective learning occurs when reliance on controlling mechanisms decreases with a shift toward automatization (Ghilardi et al., 2000; Marinelli et al., 2017). As a consequence, performance in a specific task usually improves and becomes faster and more precise (Ghilardi et al., 2000; Marinelli et al., 2017). At the neuronal level, this is usually associated with disengagement of the control-related brain areas and, in parallel, with an increased engagement of the areas that were selectively involved in the specific task (Ghilardi et al., 2000; Marinelli et al., 2017) and likely with relative growth of local synaptic weight. In this scenario, if the activity of these areas is needed in a different and successive test, delayed or attenuated spiking responses of individual cortical neurons expressed by wake local slower theta activity might occur because these neurons are already fully committed and cannot be further engaged, thus inducing performance lapses. This has been directly shown by the results of intracranial recording studies in awake humans during extended wakefulness where local, regionally specific, increases of frequencies lower than 10 Hz of the depth EEG corresponded to degraded single unit activity, increased theta activity of the surface EEG and performance errors (Nir et al., 2017). It is thus plausible that performance in the well-practiced VSEQ improved across blocks, while errors increased in *mem*, the test that shared visual working memory processes and neural basis with VSEQ. Finally, the fact that there were no notable changes across blocks in the performance of a motor test that relied more on somatosensory and motor circuits further suggests that performance deterioration is test- and task-specific.

Altogether, the present results indicate that extended wakefulness or sleep deprivation are not necessary conditions for performance decline, as continuous practice in a visual working memory task, VSEQ, in well rested young subjects can cause not only local theta power increases but also performance deterioration in a test, *mem*, that shares similarities with the task.

### A 90-min Nap Renormalizes sEEG and Improves the Ability to Learn

Our study shows that a nap restored *mem* performance and increased VSEQ learning ability, in agreement with studies showing that that even a short period of sleep improve learning indices (Mednick et al., 2003; Huber et al., 2004; Kvint et al., 2011; Lo et al., 2014; van Schalkwijk et al., 2017) and renormalized the local increase of theta power induced by extensive learning in agreement with previous studies (Nelson et al., 2021). Although quiet wake could be considered as a hybrid condition, since some of the participants assigned to this condition briefly reached N1 and N2 stages, we did not find either significant performance improvement or sEEG renormalization in the quiet wake group. In addition, we found that post-nap performance improvements were related to the nap characteristics and, in particular, strong correlation was found with low frequency power during N3 stage, but not when measured in N2 and N3 combined. Thus, it is possible that low frequency activity in N3 may be fundamental for performance improvement, although it is difficult to draw firm conclusions because of the small sample examined and the limited time of the nap. Nevertheless, N3 stage seems to play an important role in memory consolidation (Rasch et al., 2007; Diekelmann et al., 2009; Scullin, 2013) for motor learning (Huber et al., 2004), but also for other type of learning (Stickgold et al., 2000; Suzuki et al., 2012), including episodic memory tasks (Harand et al., 2012). Specifically, improvement in episodic memory correlated with hippocampal activity during N3 (Harand et al., 2012). Thus, the relationship we found between low frequency power in N3 and post-nap performance improvement suggests the occurrence during N3 of a process of renormalization in areas connected to the hippocampal circuit (Sederberg et al., 2003; Pignatelli et al., 2012; White et al., 2013).

In summary, the present findings of decreased low frequency power after a nap with improved VSEQ learning rate and performance restoration in *mem* are in agreement with the results obtained in subjects that took a nap after extensive learning with a visuo-motor task (Nelson et al., 2021). The need for sleep to restore EEG and performance further suggests that such changes were triggered by the cellular costs of increased synaptic strength induced by learning above and beyond the costs of neuronal activity *per se*, consistent with previous work (Hung et al., 2013; Tononi and Cirelli, 2014; Bernardi et al., 2015; Nelson et al., 2021).

## Data Availability Statement

The original contributions presented in the study are included in the article/Supplementary Material, further inquiries can be directed to MG, lice.mg79@gmail.com.

## Ethics Statement

The studies involving human participants were reviewed and approved by the CUNY Institutional Review Board. The patients/participants provided their written informed consent to participate in this study.

## Author Contributions

GT, CC, and MG designed the study. SR, ET, AN, PP, and HC ran the experiments. SR, ET, AN, and MG analyzed the data. SR, GT, CC, and MG wrote the manuscript. All authors contributed to the article and approved the submitted version.

## Conflict of Interest

The authors declare that the research was conducted in the absence of any commercial or financial relationships that could be construed as a potential conflict of interest.
