# Supplementary material for: Extended Visual Sequence Learning Leaves a Local Trace in the Spontaneous EEG
Source: Front Neurosci. 2021 Jul 16;15:707828. doi: 10.3389/fnins.2021.707828 (PMC8322764; doi:10.3389/fnins.2021.707828)
Supplement: Supplementary file 1 [file Table_1.DOCX]

Supplementary Material

# Supplementary Methods

**VSEQ 1 and *mem* test time-frequency analysis** For both VSEQ1 and the *mem*0 test, time-frequency representations (TFR) were computed using Complex Morlet Wavelets at linearly spaced frequencies (0.5 Hz bins) from 1 to 55 Hz. The number of wavelets cycles changes from 3 to 10, as a function of frequency. The epoch length used was 2 s portioned into 25-ms points (500-ms before to 1500-ms after the target onset). Each trial, channel, frequency, and time combination was normalized by the average of the whole time-window (1 s for mem0 and 1.5 s for VSEQ).

# Supplementary Figures


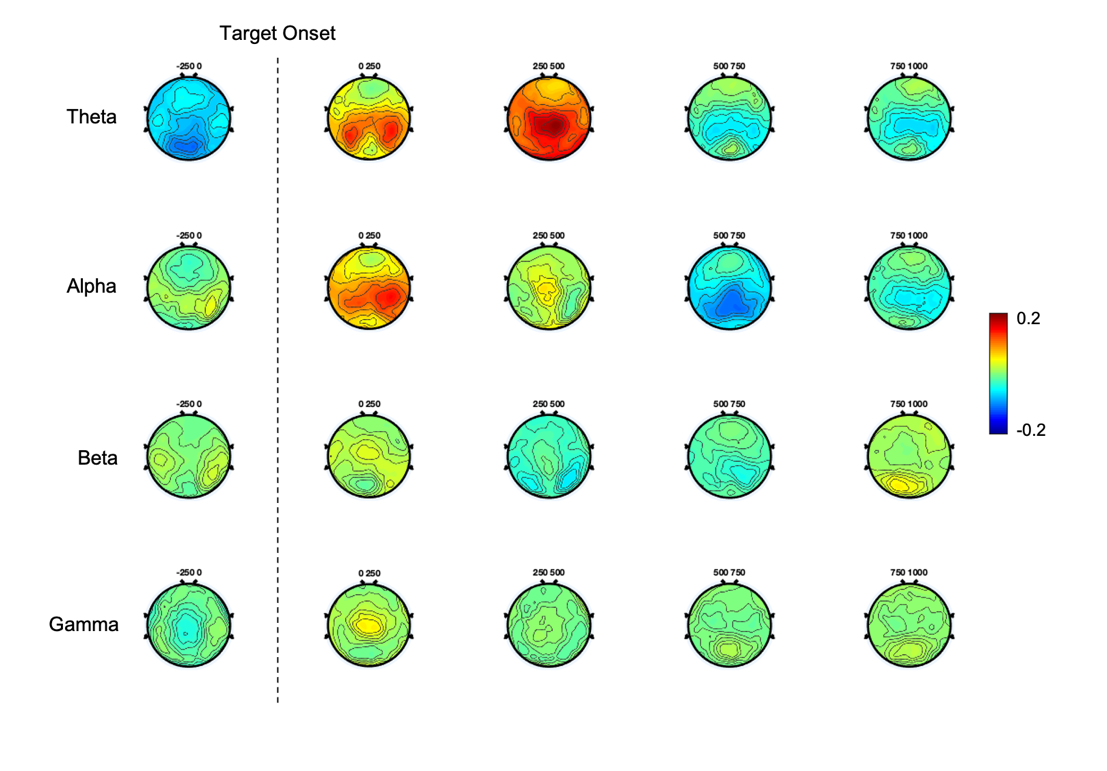


**Supplementary Figure 1.** Topographic distribution of theta, alpha, beta, and gamma oscillatory activity during VSEQ 1 in five 250-ms temporal windows starting 250 ms prior to the target appearance up to 1 s after it. From the plot it is visible a theta and alpha power increase immediately after the target appearance over a bilateral occipito-parietal region. Then, the theta power shows a more general power increase affecting also the frontal region

**
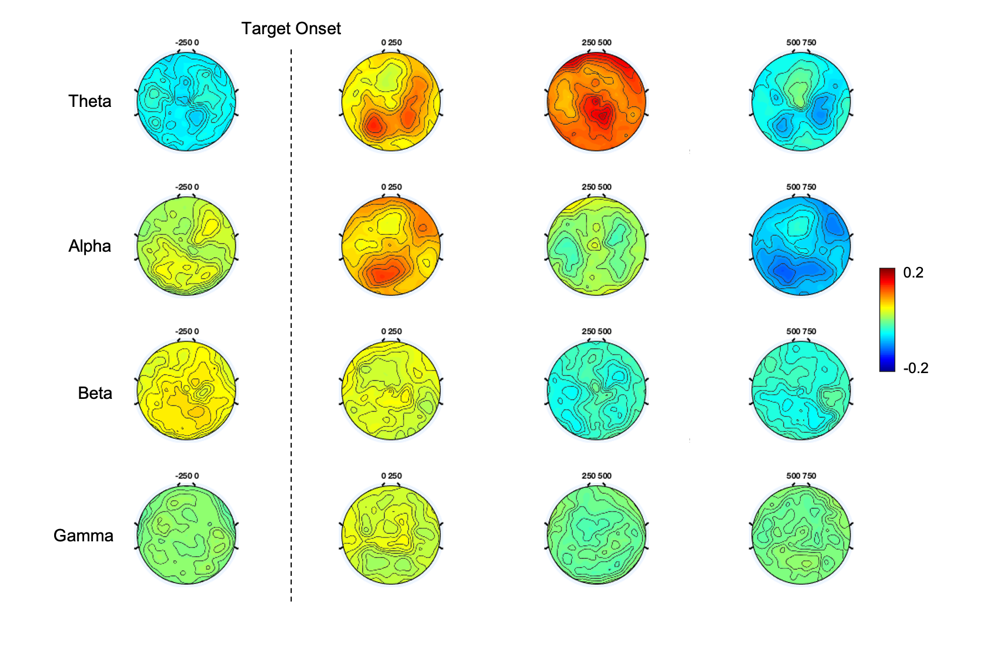
**

**Supplementary Figure 2.** Topographic distribution of theta, alpha, beta, and gamma oscillatory activity during mem 0 in four 250-ms temporal windows starting 250 ms prior to the target appearance up to 750 s after it. As for VSEQ1, the theta and alpha activity increases following the target appearance over an occipito-parietal region. The theta power further increases over a broader region. These patterns further support the idea that both VSEQ and mem recruit the same neural circuits


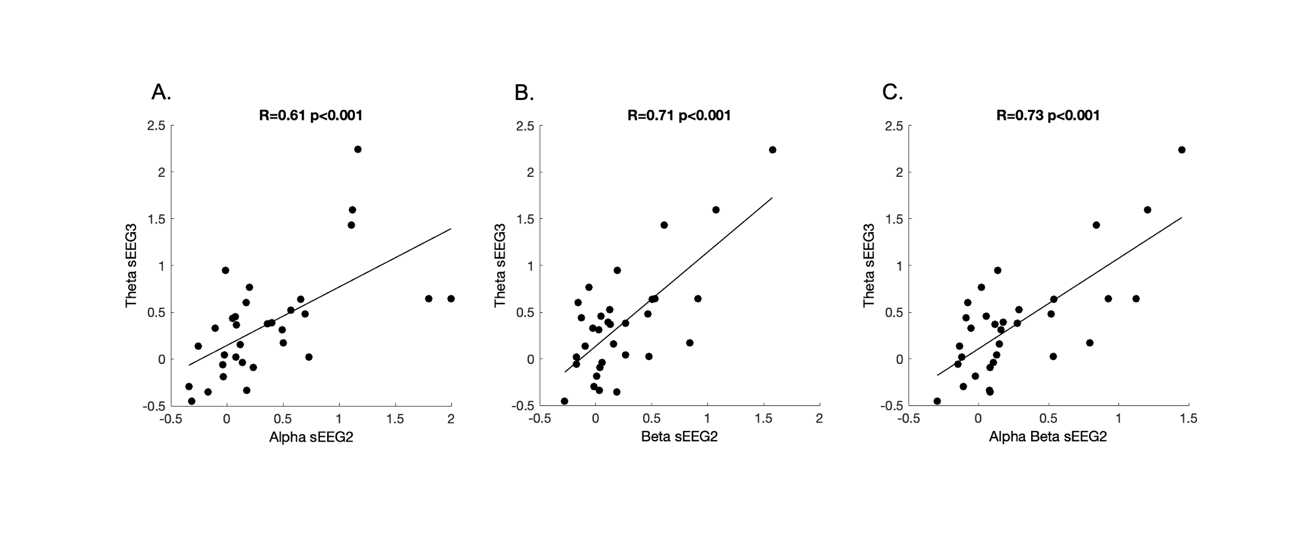


**Supplementary Figure 3.** Correlations between sEEG after VSEQ2 and VSEQ3. Theta activity recorded at the end of the morning (sEEG3) over the right temporo-parietal cluster of electrodes was related to alpha (A), and beta (B) and alpha/beta (C) sEEG2 activity over the same area

# Supplementary Tables

| **Sleep State** | **Nap**  Mean (S.E.) | **Quiet Wake**  Mean (S.E.) |
| --- | --- | --- |
| Total sleep time | 56.11 (6.31) | 24.15 (4/30) |
| N1 | 17.39 (3.07) 12/12 | 17.03 (2.40) 8/12 |
| N2 | 28.89 (4.36) 12/12 | 6.44 (2.19) 4/12 |
| N3 | 9.86 (3.18) 8/12 | -- |
| Sleep latency | 10.36 (3.20) | -- |
| N2+N3 | 38.75 (6.43) | -- |

**Supplementary Table 1.** Sleep stages duration (minutes). The table indicates the duration of different stages of sleep in the nap and quiet wake groups (mean and standard error of the mean). Sleep latency is the first occurrence of N1.

|  | **Global Power** Mean (S.E.) | **Right ROI**  Mean (S.E.) |
| --- | --- | --- |
| **Normalized N2** |  |  |
| Delta | 2.96 (0.26) | 2.78 (0.25) |
| Theta | 1.69 (0.12) | 1.73 (0.14) |
| Alpha | 1.74 (0.11) | 1.60 (0.13) |
| Spindles (12-16 Hz) | 2.19 (0.19) | 2.10 (0.22) |
| Beta | 1.21 (0.06) | 1.21 (0.08) |
|  |  |  |
| **Normalized N3** |  |  |
| Delta | 7.89 (0.91) | 8.60 (1.38) |
| Theta | 2.29 (0.15) | 2.24 (0.18) |
| Alpha | 1.81 (0.13) | 1.56 (0.31) |
| Spindle | 1.86 (0.14) | 1.74 (0.28) |
| Beta | 0.79 (0.07) | 0.80 (0.11) |
|  |  |  |
| **Normalized NREM** |  |  |
| Delta | 4.13 (0.62) | 4.17 (0.72) |
| Theta | 1.83 (0.13) | 1.85 (0.14) |
| Alpha | 1.76 (0.11) | 1.60 (0.15) |
| Spindle | 2.13 (0.19) | 2.02 (0.22) |
| Beta | 1.14 (0.08) | 1.13 (0.09) |
|  |  |  |
| **Normalized SWE** | 2.16 (0.67) | 2.28 (0.74) |

**Supplementary Table 2.** Sleep characteristics in the nap condition. Global mean spectra for the five frequency bands during artifact free EEG normalized by N1 power for the given band. NREM is the mean power during N2 and N3 sleep. Slow wave energy (SWE) is the mean normalized delta power of N2 and N3 multiplied by their respective durations
